# Towards a post-age picturebook pedagogy

**DOI:** 10.1057/s41599-022-01446-4

**Published:** 2022-11-29

**Authors:** Xiaofei Shi

**Affiliations:** Soochow University, Suzhou, China

**Keywords:** Literature, Education

## Abstract

This article is one of the first attempts to materialise Joanna Haynes and Karin Murris’ conceptualisation of the post-age pedagogy that focuses on loosening the boundaries of age-based expectation. The project is conducted against the backdrop of the discourse of ageism existing hand-in-hand with various age-transgressive practices. It involved multiple case studies of child and adult readers’ responses to two picturebooks, i.e., *How to Five Forever* (1995) and *Grandpa Green* (2011). The two research questions are: how do children and adults respond to *Forever* and *Grandpa*? What is the pattern of similarities and differences in their responses? In addressing these research questions, the present study aims to facilitate the emergence of a post-age picturebook pedagogy. The findings reveal that a post-age picturebook pedagogy should acknowledge the chance of holistic learning opportunities that picturebooks can offer to both children and adults, including, for instance, aesthetic appreciation, literary understanding, emotional engagement, and material experience. Such a pedagogy involves an egalitarian view of child and adult that acknowledges some biologically determined differences subject to the factor of age and meanwhile emphasises their shared humanity and idiosyncrasies, thus undermining the binary division between simple child and sophisticated adult. The material affordances of a picturebook can be deployed to amplify the points of connection and dialogue in child and adult readers’ responses and a post-age picturebook pedagogy should strive to enrich interpretive and experiential possibilities for diverse readerships. In comparison with the existing empirical studies using picturebooks, this project positions adults as readers who can enjoy picturebooks for their own sake rather than co-readers whose responsibility is mainly to facilitate child readers’ learning and development. It stresses and seeks to maximise the potential of picturebooks for creating a beneficial and pleasant experience for all involved, both child and adult, an aspect that has long been overlooked.

## Introduction

We are living in a contradictory world, in which, on the one hand, there are many kinds of age-transgressive practices and on the other hand, ageism that rests upon rigid correspondences between a person’s age and their status, abilities, and behaviours characterises various discourses. This sort of contradiction is epitomised in the field of children’s literature. This field itself is made possible by the age-based distinction, i.e., children are younger than adults and thus, they need, or rather, they are considered to need a separate kind of literature specially written for them. However, it is not uncommon to see children reading books for adults and vice versa, and the reader’s crossing of the boundaries between children’s literature and adult literature is called cross-reading (Falconer, 2004). Correspondingly, “cross-writing” is used to denote the phenomenon that a writer may target different readerships with their different works (Beckett, 1999), and some of the most famous writers who cross-write include, for instance, C.S. Lewis, J.R.R. Tolkien, and Michel Tournier. Moreover, the wide popularity of the *Harry Potter* series gives rise to the term “crossover literature” (Beckett, 2009; Falconer, 2009; Hoffmann, 2018; Shi, 2022), which refers to a group of texts that can maintain an attraction for both children and adults. Thus, today’s field of children’s literature abounds in a variety of age-transgressive practices.

Contemporary age-transgressive practices in children’s literature are not limited to some isolated cases of popular books but situated in the social milieu in which “child and adult cultures are clashing, intersecting and hybridising” (Falconer, 2009, p. 3) due to globalisation (Prout, 2005) and developments in digital technologies and social media (Harju, 2012). Moreover, today’s world witnesses a drastic shift to the pictogram mode, which facilitates narrative forms that heavily rely on the visual, such as picturebooks, to extend to diverse readerships. Since W.J.T. Mitchell (1994) coined the notion “pictorial turn” to describe a growing interest in the visual, high-speed connections, the almost instantaneous appearance of image-laden websites, the invention of smartphones and tablets, and image-focused social networks, all of them being enabled by information technology, have helped to create a “near-constant stream of images” (Shiel, 2013, p. 77). Images are even posing a challenge to the status of writing as the central mode of representation in education, as learning resources are increasingly distributed through digital media rather than traditional (text)books (Bezemer and Kress, 2008). Burgeoning livestream learning and teaching in the wake of the COVID-19 pandemic further contribute to the ubiquity of images. Thus, it is not surprising to see adults turning to picturebooks that were traditionally exclusively associated with children.

Meanwhile, there is an undercurrent of ageism beneath some important discussions on contemporary children’s literature. Words such as “infantilisation” and “infantilism” reflect a deep-seated anxiety about adults reading books for children, and this concern has been voiced by prominent literary critics like Harold Bloom and A.S. Byatt, as Rachel Falconer demonstrates (2009, p. 43). The line of argument underlying such voices is that children’s books are too simple and therefore too degrading to be read by thinking adults. One way to combat the critique of infantilising adults that has often been levelled at contemporary children’s literature is to demonstrate that children’s literature is not simple but rather complicated, with its discussion of controversial topics, such as violence, sex and death, and its incorporation of postmodern characteristics and devices. However, this may result in another kind of critique—the adultisation of children, as illustrated by Sandra Beckett (2009, p. 258). In fact, underlying these two types of concern is the same mode of thinking which assumes that a norm exists for being a child or adult so that any deviation from this norm should be decried as premature or degrading.

Indeed, a lot of attempts have been made to build a scientific groundwork for learning and teaching that takes into account the factor of age. For instance, Kurt W. Fischer suggests that people move along a continuum of actions, representations and abstractions between birth and early adulthood and as people develop through these stages, brain activity reorganises itself, forming new neural pathways and networks (2009, pp. 10–11). At the same time, cognitive studies on reading development have also questioned strict stage models of comprehension. Rather, they start to recognise multi-component models; the coordinated activity of reading involves various kinds of knowledge and skills, including, for instance, vocabulary, syntactic knowledge, inference-making, working memory, and metacognition (Paris and Hamilton, 2009, pp. 38–45). Moreover, the knowledge and skills involved in reading follow different developmental trajectories, varying “widely in age of onset, duration and rate of learning, and level of proficiency and mastery” (p. 39). Thus, the present study remains wary of the strictly age-based categorisation of books and readerships.

This project is conducted against the backdrop of ageism that exists hand-in-hand with age-transgressive practices in the field of children’s literature. Picturebooks, as a key subcategory of children’s literature that relies on the interaction between verbal and visual narrations, used to be stigmatised as naïve readings that children will grow out of once they learn to read chapter books. Yet picturebooks are attracting both children and adults these days (Beckett, 2012; Kümmerling-Meibauer, 2015; Ommundsen, 2015; Shi, 2022). Thus, the conflict between ageism and age-transgressive practices may become more intensified in the area of picturebooks compared with other forms of children’s literature. Following the step of Joanna Haynes and Karin Murris who envision a post-age pedagogy as a thought experiment to loosen the boundaries of age-based expectation (2017), this project aims to provide some tentative thoughts that may allow a post-age picturebook pedagogy to take shape through a case study of child and adult readers’ responses to picturebooks.

## Literature review

Picturebooks have been acknowledged as a highly valuable medium for child education. Evelyn Arizpe suggests that crucial to using picturebooks in an educational context is a careful consideration of “all the affordances of a picturebook” that can create “holistic opportunities for learning” (2021, p. 268). Existing studies on picturebooks in child education revolve around four key dimensions of learning opportunities, i.e., cognitive, affective, aesthetic, and material. The cognitive aspect of a picturebook’s affordances include, for instance, the acquisition of language, conceptual domains, moral concepts and multimodal literacy skills, and the forming of abilities related to space and time (Flint and Adams, 2018; Kümmerling-Meibauer, 2013; Pantaleo, 2012, 2013; Silva-Díaz, 2015). The affective affordances of a picturebook are about evoking readers' emotional engagement, such as identification and empathy (Nikolajeva, 2014). Formal elements like page layout, typography, frames and endpapers direct readers’ attention to the picturebook as an aesthetic artefact (Lambert, 2018; Pantaleo, 2012; Sipe and McGuire, 2006; Smith, 2009), which may pave the way for future literary understanding (Sipe, 2008; Zapata et al., 2018). Compared with the other three kinds of affordances, material affordances receive relatively less academic attention. Books are physical entities and for picturebooks, physicality plays a more significant role, as many picturebooks, for example, pop-up ones, deliberately play with physical elements of a book. Thus, the material affordances of a picturebook may give rise to “physical, multisensory, and interactive” experiences of reading (Arizpe, 2021, p. 262).

The various uses that picturebooks can be put to in child education aside, this narrative form is also attracting widening readerships that include adults. Haynes and Murris note that “contemporary picturebook artists have long since breached the confinement of picturebooks to a particular age group and the genre has become increasingly sophisticated and taken more seriously” (2017, p. 3). Beckett devotes a monograph to the discussion of what she calls “crossover picturebooks”, by which she means those picturebooks that transcend the boundaries between traditional children’s literature and adult literature (2012). In Scandinavian countries, there emerges a group of picturebooks that addresses issues probably of more interest to adults than to children, such as existential enquiry, abortion, suicide and so on (Ommundsen, 2015). Bettina Kümmerling-Meibauer considers the phenomenon of picturebooks reaching other readerships than children as an important characteristic of the contemporary European book market (2015). Pioneering studies have just started to test the use of picturebooks in various institutions ranging from kindergarten to university (Ommundsen et al., 2021).

The next issue arises as to how a widening range of picturebook readerships may inform the picturebook pedagogy. Beckett emphasises that picturebooks, with their “universal appeal” (2012, p. 111), can be “a genre for all ages” (2012, book title). Statements as such stress the potential of picturebooks for attracting children and adults at the expense of flattening the diversity in readers and their possible differences subject to the factor of age. Other scholars have taken a different approach, suggesting that picturebooks are for children if read in an unsophisticated way yet some may also address adults who enjoy untangling the complicated web of intertextual references (Desmet, 2004). Claims made along this line indeed acknowledge possible differences in readers, yet underneath is a binary division between simple child and sophisticated adult. Moreover, in contrast to a vast number of empirical studies using picturebooks with children, those involving adults remain scarce. In the few projects that involve adults as picturebook readers, adults are mostly positioned as facilitators in situations of shared reading to scaffold children’s learning and development (e.g., Bus and Van Ljzendoorn, 1997; Muhinyi and Hesketh, 2017). In other words, it seems as if adults could not read, let alone take pleasure in, picturebooks except as parents, teachers, librarians or publishers.

Thus, the new developments in the field of picturebooks that help to extend the appeal of this narrative form to an unprecedentedly wide range of readerships call for a rethinking of the picturebook pedagogy. This task becomes urgent, given that much less academic attention has been paid to adults as picturebook readers for their own benefit and pleasure.

## Methodology

### Key conceptual understandings

Of crucial importance to reformulating the picturebook pedagogy in light of its widening readerships is how to conceptualise child and adult. A post-age pedagogy is to challenge and even subvert a linear understanding of life and rigid age categorisations (Burman, 2008; Haynes and Murris, 2017; Lee and Motzkau, 2011). However, age is much more than a label. Rather, it is a constituting factor of what we may think of ourselves, what others may think of us, and who we are. Even Haynes and Murris acknowledge that a complete post-age pedagogy may not be possible: “[o]ur theorising has generated some improbable and provocative ideas that are unlikely to find their way into the current target driven education policy discourse but appeal to our philosophical imagination” (2017, p. 11). Nevertheless, a quasi-post-age pedagogy may take shape by reflecting on conceptions of child and adult, two largest age-based categories, as a starting point.

It is here that Marah Gubar’s kinship model (2016) comes in handy. Arguing against idealising or demonising the child (which, according to Gubar, features the difference model of childhood) and treating the child as inferior to the adult (which features the deficit model), Gubar proposes the kinship model. The kinship model encompasses three dimensions—some biologically determined differences between child and adult, their shared basis of humanity, and the possibility of idiosyncrasies due to neurodiversity and social conditions (Gubar, 2016, pp. 299–300). The kinship model informs how this project conceives child and adult readers.

Moreover, cognitive literary studies, which combine findings from developmental psychology and neuroscience with literary research, have provided valuable insights into what the three dimensions of the kinship model, in relation to literary reading, maybe about. First of all, there may be age-linked differences in literary engagement. For instance, there is a stable correlation between development in metacognition, the knowledge and control of one’s own cognitive processing, and reading comprehension performance (Paris and Hamilton, 2009). Metacognition only starts to play a major role in mental activities in the early primary years (Goswami, 2011, p. 646). Thus, early readers often demonstrate little comprehension monitoring (Baker and Beall, 2009) and young children are more likely to focus on word decoding than evaluating their understanding of a text (Johnston and Afflerbach, 1985).

Despite some biologically determined differences subject to the factor of age, we are all subjects “under the big tent ‘People’” (Gubar, 2016, p. 295). Thus, there must be some mechanisms and activities shared over the life course. Of particular concern for picturebook reading is conceptual blending. According to Gilles Fauconnier and Mark Turner in their ground-breaking work *The Way We Think* (2002), human beings can activate two conflicting mental structures and creatively blend them into another emergent structure. Turner then proposes the notion of a double-scope story, in which input stories with different organising frames are blended into a third story, hence giving rise to new meanings (2003, p. 128). Double-scope stories can be found almost everywhere, from picturebooks for babies to the religious narrative of Christ the Redeemer (Turner, 2003, pp. 117–142). The picturebook by its form invites readers to synthesise the input story narrated in the verbal and that narrated in the visual so as to create a blended story with new meanings.

Growing up defies a one-size-fits-all pattern. Even developmental psychologists who focus on limitations and differences due to the factor of age acknowledge individuality in cognitive development. For instance, Robert J. Sternberg states that cognitive skills are modifiable depending on the environment (2011, p. 759). As Gubar puts it, “[g]eneralizing about what powers and abilities children do (or do not) have is problematic because children, like adults, are a heterogeneous bunch” (2016, p. 294). Maria Nikolajeva stresses that some skills necessary for literary engagement may not be age-related, for example, the skill of making connections to life and literary experiences (2014, p. 16). After all, readers are idiosyncratic beings immersed in different environments and exposed to various literary, artistic and cultural works.

The degree of differences between child and adult may depend on maturational limitations and individual propensities. Still taking metacognition as an example, a dramatic increase in metacognition often takes place between three and four years old (Lomax and McGee, 1987) while brain changes over adolescence continue to exert a significant influence on one’s skill of planning, evaluating, and self-regulation (Levitin, 2006). Thus, presumably in general, there might be greater differences in the development of metacognition between infancy and adolescence than within the category of adolescence itself. However, given the possibility of atypical development in metacognition and variations in activity or experience that may enhance or inhibit metacognition, there might be greater differences within the category of adolescence than between adolescence and adulthood.

Therefore, the conception of child and adult readers that encompasses some biologically determined differences, shared humanity and idiosyncrasies is important for taking the first step on the way toward developing a post-age picturebook pedagogy.

### Texts

Two key considerations for text selection are theme and artistic style, which, ideally, should combine to create textual affordances that may cater to both children and adults. Accordingly, Colin Thompson’s *How to Live Forever* (1995) (henceforth *Forever*) and Lane Smith’s *Grandpa Green* (2011) (henceforth *Grandpa*) are chosen. Both picturebooks deal with the theme of mortality. In *Forever*, Peter does not want to grow old and embarks on a journey of seeking the one and only book containing the secret of living forever. After meeting the Ancient Child who read the secret book and has been arrested in time since then, Peter relinquishes the idea of immortality. *Grandpa* tells the story of a little boy who relives through his great-grandfather’s life experiences that have been carved into topiary images. The great-grandfather, the creator of the topiary garden, passes away in the end and the little boy inherits the art of horticulture. Thematically, both picturebooks concern how human beings come to terms with mortality. Beckett sees death as a cross-generational theme because it is relevant to all human beings (Beckett, 2012, p. 249). As Hugh Crago observes, even pre-schoolers may become distressed at the sight of damaged objects because though they may not be able to recognise or articulate their distress or fear, the damage triggers an “existential anxiety—a sense of the vulnerability of the child’s own body” (2014, p. 39). Therefore, in terms of theme, the two picturebooks may resonate with both children and adults.

Despite the thematic similarity, *Forever* and *Grandpa* contrast in style. First, the verbal text in *Forever* is denser than that in *Grandpa*. The former contains quite a few lines of words per page, while in the latter, usually there are only one to two sentences per page. Second, the pictures in *Grandpa* combine watercolour and pencil strokes, resulting in an elliptical style, while those in *Forever* depict multitudinous miniscenes in a realist style. Third, the verbal–visual relationship in *Grandpa* is more varied than that in *Forever*. In *Grandpa*, the words can appear in the blank space or be overlaid with the pictures. Often, one sentence runs over two pages. The pictures can complement, enhance or counterpoint the words. The words in *Forever* are often framed off from the pictures. The pictures mostly complement or enhance the words. Such thematically similar yet stylistically contrastive books may generate interesting data about how nuanced aspects of a picturebook may affect its appeal to children and adults.

Based on the preliminary work of text selection, the study sets up two research questions: How do children and adults respond to *Forever* and *Grandpa*? What is the pattern of similarities and differences in their responses?

### Readers

The project involved multiple case studies of children and adults. The research was set in a small town in the east of England with several academic institutions in its surroundings, which possibly indicates an above-average level of education of the residents and hence a relatively high reading literacy level of the participants. A purposeful sampling strategy was used. The call for participants emphasised two main factors, i.e., basic literacy skills and prior reading experience, and specified that both children and adults were welcome. The volunteers’ self-report was taken into consideration, especially that of their ability to read and communicate in English and to handle some basics of visual literacy, including, for instance, what images may signify, since the selected primary texts were picturebooks written in English. The participant call was circulated through the institutions’ mailing lists and made into a poster put up at the city centre.

Among all the six people who responded to the call for participants, only a valid sample of four remained throughout the project. Of the two who dropped out halfway through, one participant moved out of the area in which the research was conducted for personal reasons, while the other had an urgent task at work that required more focused attention. The final valid sample included two children (aged 8 and 9) and two adults (aged 24 and 30), with only one male (the younger child). Oliver (male, 8 years old) is from Ireland and was studying at a local primary school. He acknowledged that he was not a big fan of reading and when he read, he preferred comic books. Lucy (female, 9 years old), also from Ireland, was studying at the same local primary school as Oliver. She was extremely capable of expressing herself, well-read, and on the point of perceiving picturebooks for younger readers. Rihanna (female, 24 years old) is American and was doing a Master’s degree. She had been a schoolteacher for two years, working with different age groups. She had taken a short-term course in children’s literature, which was designed to train teachers. Sophie (female, 30 years old) is Chinese and was studying at the doctoral level. She was well-read and had been painting.

Therefore, the collective case study had four cases, with one reader being one case. The collective case study was instrumental (Stake, 1995) in that four cases were compared for patterns of similarities and differences that would inform the development of a post-age picturebook pedagogy. The differences in the educational and cultural backgrounds of the participants coming from various countries would ideally help to generate multiple perspectives, which are crucial for a collective case study (Creswell, 2012; Creswell and Poth, 2017). Such diversity in backgrounds was also desirable for this project because diversity features real-life situations of reading. Constructivist approaches to reading as a meaning-making activity (e.g., Langer, 1995) emphasise that the background, history, knowledge and experience of a reader impact both the processes and products of reading. Thus, including the four participants from diverse backgrounds reflects the attempt of this project to accommodate the potential diversity in real reading scenarios. Moreover, since the present study aims to explore the patterns of similarities and differences in the four readers’ responses so that some preliminary ideas regarding a post-age picturebook pedagogy can emerge, it generates no evaluative conclusions to rank their responses.

### Reading

Each participant was to read both *Forever* and *Grandpa* and for each picturebook, there were two encounters. In the first encounter, I encouraged the participants to talk about salient textual aspects that might particularly attract them. The second encounter was a follow-up from the first one, picking up prominent issues and offering an avenue of more in-depth discussion. The second encounter often generated more condensed elaboration on parts of the picturebook that the readers were most interested in. Therefore, four sessions of the semi-structured interviews were carried out separately with individual participants. The choice of individual interviews arose from the concern that this project considers adults more as readers who can enjoy picturebooks for their own sake than as co-readers to facilitate children’s learning and development. The interviews contained a series of open-ended questions about what stood out most to the readers (see Appendix I for the interview prompts) and I remained open to unexpected issues and ideas that they put forward. All interviews were videotaped.

The participants were encouraged to talk about what they had in mind at the same time of reading (i.e., the think-aloud method) or they could also choose self-probed retrospection. In self-probed retrospection, readers make marginal marks rather than speak out whenever textual clues conjure up anything in their minds. When the reading is over, readers are asked to describe what they are reminded of at each marking place (Larsen and Seilman, 1988). Self-probed retrospection, as an alternative to the think-aloud method, can be less disruptive and embarrassing (Miall, 2006). The interviews started with the instruction that encouraged the readers to take their time, stay on the pages at their own paces, and share at the same time of reading anything that caught their attention. Then I observed the readers’ reaction and asked whether they were fine with this. If they showed any sign of reluctance, I would provide them with colourful pens and post-its and add that if they were not comfortable with talking while reading, they could paste the marked post-its on relevant pages and talk about these pages after they finished reading.

### Data analysis

The videotaped reading sessions were fully transcribed. Data analysis underwent three stages. In the first stage of within-case analysis, I investigated how each reader engaged with the two picturebooks, using a coding scheme that emerged from the set of data itself through a grounded approach (see Appendix II for the coding scheme). In the second stage, a cross-case analysis was conducted, focusing on what aspects of the two picturebooks featured prominently in all the readers’ responses. These aspects included setting, narrative structure, and intertextuality. In the last stage, I compared and analysed across the cases to reveal the patterns of similarities and differences in the four readers’ responses to the same textual aspects of the two picturebooks.

## Results

### The world of books

*Forever* portrays a fictional world full of books. Most of the settings depicted in the pictures have something to do with books, including, for instance, a library, file cabinets, and bookshelves. The crevices of these places are lighted as if they were inhabited by miniature people. Moreover, the settings are tightly packed with enigmatic visual details and verbal jokes. Limbs seem to act on their own, dilapidated odds and ends lie around, and book titles poke fun at well-known classics and cultural conventions. The four readers spent a lot of time naming, pointing at, and sorting through the multitudinous visual details, and they all attempted to fill the textual gaps that they had spotted yet in different manners.

One of the textual gaps that featured prominently in their responses was how to interpret the lighted world of books. There were two ways to fill this gap—one was more literal, which suggested that books were an actual space, functioning as the home for miniature inhabitants, while the other was more elevated, which saw books as an abstract space of knowledge and life stories. The child readers demonstrated the literal reading and the adults manifested the elevated reading.

The contrast in this aspect between the children’s responses and the adults’ can be revealed by examining the following examples:

maybe they can live all up inside the whole shelf, oh ~ [clapping]. Ba, ba, ba, ba [laughing, and emulating how a person goes up with her fingers]. It could be like a block of apartments, like there’s a room there, a room there, and a room there [pointing at the books all the way up the bookshelf]. And somebody may live in the ceiling, between the ceiling and the floor, there’s a book, pull it out [pretending to pull something down from the ceiling], that would be so cool [laughing and waving her hand in a big circle]! It would be awesome to live in a shelf [laughing and pretending to snatch a book and read]. Snatch, Snatch [laughing and pretending to snatch hard]. It’s great! And then you’ve got a front door and a back door. In the front door, out the back door, go there, so like bye bye, Amanda, hello, Samantha [laughing]! They have a great view over the city! (Lucy)

sometimes he just imagines, “oh, what if I can live in the books to read all day.” So either it’s a dream or it’s what happens to him because he has this wish to live among all those books. He came into this place and then had a real experience of what it would be like to live among all those books. But living among the books, it turned out to be not that enjoyable [laughing]. So he realises that eventually he has to live in the reality, back in the reality [pointing at the last page]. (Sophie)

In the parallel of the examples above, Lucy fleshed out a bustling world of books, where miniature people lived all the way up the bookshelf, winding their way around, greeting friends, and snatching books as they climbed up. Lucy’s gesture aligned seamlessly with her speech to convey the light movement of the miniature inhabitants and the enormous effort that it took them to snatch books from the bookshelf. Moreover, her narrative unfolded in a rhythmic flow, for instance, “a room there/and a room there/snatch/snatch/in the front door/out the back door/bye bye Amanda/hello Samantha”. Iain McGilchrist observes that the musical aspects of language (such as intonation, rhythm, and pitch) develop in children before syntax and vocabulary (2012, pp. 103–104). This echoes Crago’s finding that children, younger than 2 years old, can experiment with baby talk of a musical nature (2014). The meaning of baby talk lies more in its sound patterns than in the words used (Crago, 2014, pp. 15–17). McGilchrist even suggests that music is the “communication of emotion”: “the prosody and rhythmic motion … emerge intuitively from entrainment of the body in emotional expression” (2012, p. 103). Lucy, while imagining herself living through the fictional world, had a variety of bodily experiences—climbing up, waving, anchoring herself with clawing hands, and sparing one hand to snatch a book, which gave rise to the musical aspects of her narrative and its nuanced emotions, including, for instance, joy at seeing and greeting friends and excitement at taking in a whole view of the city when one finally arrived at the top.

In comparison, Sophie traced the movement of Peter through various settings, keen on identifying a thread that would string together multitudinous visual details. She criticised the book’s “dry” verbal narration and turned to seek what kind of story she would make just by focusing on the illustrations. In her story, the protagonist Peter entered the bizarre world of books because he was enthusiastic about book knowledge. Sophie’s narrative engaged the schema for SOURCE-PATH-GOAL: Peter started out with the fancy of living in books and a series of adventures took him through various book worlds until he finally reached a new understanding of the relationship between the reality and book knowledge—book knowledge could never replace one’s experience of the reality. Interestingly, despite her critique of Thompson’s storyline, Sophie came up with one that shared an underlying embodiment of the inner quest in one’s physical movement. Embodiment, which means that our understanding of abstract concepts is rooted in our bodily movement, is “an essential part of the perceptual and cognitive processes by which we make sense of our experiences in the world” (Gibbs, 2005, p. 3). To take the function of image schemas as an example, image schemas are generally defined as “dynamic analog representations of spatial relations and movements in space” (Gibbs, 2005, p. 90). Image schemas not only organise our bodily experience but feature in our thinking and imagination (Johnson, 1987). While embodiment featured in Lucy’s responses as an ensemble of speech, gesture and rhythm, for Sophie, embodiment was manifest in her using the SOURCE-PATH-GOAL schema to discuss the abstract notion of a quest.

### The blended world

*Grandpa* stands out as a double-scope story. First, the topiary images blend two conceptual domains, namely, tree and life experience. Moreover, the garden of topiary images that the great-grandfather creates constitutes a blended world: it is both a physical space and an abstract domain that stands for the great-grandfather’s past, and projects the shared mindscape of the great-grandson and the great-grandfather. All the readers recognised in topiary images the blending of two conceptual domains. But the two child readers were amazingly similar in dissociating the domain of tree from that of life when the picturebook depicts the little boy kissing a topiary girl in arabesque with her braids flying and skirt ballooning. Oliver, for instance, let out peals of laughter, exclaiming that the little boy was actually kissing a hedge. The dissociation of two conceptual domains, in this case, happened probably because it created a humorous effect—the boy on tiptoe stole a kiss from a gigantic hedge at least twice larger than him.

As to conceptual blending in terms of the overall structure, the adults were more likely than the children to synthesise the two input stories in the visual and verbal narrations. Oliver struggled with the relationship between the protagonist “he” mentioned in the verbally narrated story and the image of the little boy portrayed in the visual. Though Lucy identified the little boy as the great-grandson of the protagonist “he”, she focused on the spatial dimension of the garden, making no mention of the space as a shared mindscape. In comparison, Rihanna not only blended the visual and verbal narrations but questioned the gap in their counterpart relations:

It’s interesting they’re [the words are] talking about him forgetting things, but elephants [pointing at the topiary elephant] are known for remembering everything. The author could have used a kangaroo, he could have used a rabbit, he could have used a dog or something. I’m curious why he chose an elephant. I wonder if it does have something to do with kind of being on the opposite side of the spectrum. The person that we’re talking about forgets things while the elephant doesn’t, I don’t know.

In this example, Rihanna presumed that there must be an authorial intention to juxtaposing the statement of the old man’s forgetfulness and the image of a topiary elephant. On the one hand, she attempted to justify the author’s choice. On the other hand, she offered her own solution—rather than put a topiary elephant here, she would have used a goldfish that was known for its “memory of two or three seconds”.

### Intertextuality

What *Forever* and *Grandpa* have in common is their use of intertextuality, i.e., the presence of text A (the intertext) in B or the interplay of identifiable texts (Moraru, 2005, pp. 256–257). *Forever* abounds in intertextual connections that play with literary classics, such as “Lord of the Pies”, “Gone with the Wine”, “The Merry Chives of Windsor” and so on. Each box of the bookshelf on the third spread^1^ is organised around one theme. The titles of the classics, such as *Lord of the Flies*, *Gone with the Wind*, and *The Merry Wives of Windsor*, are deliberately twisted to fit into the overarching theme—food and drink. The intertexts that *Grandpa* refers to were all published in the early 1900s. *The Secret Garden* first came out in 1911, *The Wonderful Wizard of Oz* in 1900, and *A Little Engine that Could* in 1930. The selection of these intertexts makes sense because the great-grandfather was born at least before WWII. The readers’ responses to the intertextual references in the two books can be sorted into passive response—confusion when they failed to identify the intended intertexts—and active response. In the latter case, two sub-types can be specified: one was identifying the intertexts and exploring how they functioned in the story world, while the other was engaging in an interpretive play in the absence of the knowledge of the intended intertexts.

Compared with the world of books and the blended world, intertextuality generated perhaps more within-category differences. While Lucy associated the pumpkin whence light is shining (*Forever*, spread 3) with the story about Cinderella because “there’s a pumpkin in *Cinderella*”, the same intertext resulted in the feeling of confusion in Oliver—“someone is living in a pumpkin. That’s so weird”. While Sophie commented on “the little engine that could” with “this sentence is incomplete”, Rihanna recognised that it referred to the eponymous picturebook first published in America in the 1930s. This makes sense, given her nationality and past working experience as a teacher. In a 2007 survey, the American National Education Association included *The Little Engine That Could* in “Teachers’ Top 100 Books for Children”.

The second sub-type of active response, i.e., interpretive play, was mostly manifest in the child readers. Shortly after temporary confusion over the incompleteness of the phrase “the little engine that could”, Oliver and Lucy creatively filled the relationship gap between *Grandpa* and the intertext as follows:

“he read stories about secret gardens and wizards and a little engine that could” [reading aloud the words on the fifth spread, and on finishing “a little engine that could”, Oliver turned to the next page to see if he had missed anything], wizards, and a little engine that could, that could, that could fly, that could fly [pointing at the image of the flying engine]! (Oliver)

I don’t know [what comes after “a little engine that could”]. Maybe a little engine that could go to school or something [laughing and pointing at the previous sentence “he had to stay home from school”]. (Lucy)

Mary-Anne Shonoda believes that intertextuality evokes a nebula of loosely connected associations rather than direct a linear relationship between primary-text and intertext, and thus the ensuing textual response is often playful and idiosyncratic (2012, p. 84). Even when the intertext was quite explicit in the case of “the little engine that could”, the children could still decide for themselves the most relevant connection in the network of associations evoked. The image of an engine cutting its way obliquely through the sky encouraged Oliver to supply the word “fly” after “the little engine that could”, while Lucy, adopting a non-linear way of reading, went back to the previous sentence to supply her choice of the missing word.

## Discussion

A post-age picturebook pedagogy should acknowledge that both children and adults can benefit from the holistic learning opportunities that a picturebook may offer, including and not limited to aesthetic appreciation, literary understanding, emotional engagement and material experience. What adults may derive from picturebooks has long been neglected. This project demonstrates that the gap created by the tension between the verbal and the visual in a picturebook can stimulate adult readers to actively use their prior life and literary experiences, for instance, in the case of Sophie retelling a story grounded in the visual settings and Rihanna suggesting another textual choice of a topiary goldfish in place of the topiary elephant. Moreover, Sophie particularly commented on how *Grandpa* impacted her emotionally—“how a person can express his feelings, how he deals with memory, and past experience, and feelings about past experience”.

With adults welcomed back into the arena of picturebook reading, then the next issue is how to conceptualise the power relationship between child and adult readers. This project demonstrates that the different ways in which children and adults fill the textual gaps cannot perhaps be fully captured by the binary division between simplicity and sophistication. The existing studies rightfully point out that some visual and verbal gaps are left to be filled differently by children and adults (e.g., Nikolajeva and Scott, 2001, p. 24). Discussions on the potential of intertextuality for addressing a wide range of readerships often involve how “complicated” intertextual connections may attract adult readers (Desmet, 2004; Beckett, 2012). Indeed, more elaborate engagement with conceptual blending at the level of the narrative structure occurred in the adult readers. However, at least in this project, it is difficult to pinpoint whose responses to intertextuality were more sophisticated or mature. Rather, the two child readers demonstrated a more creative engagement with intertextuality while the adult readers did not. It perhaps makes sense to suggest that intertextuality contributes to diverse addresses because it makes allowance for a variety of readings, depending on the reader’s cultural background, life and literary experiences. Moreover, the verbal–visual interaction in a picturebook may leave more room for non-linear readings. For instance, the image of an engine winding its way through the sky encouraged Oliver to supply the word “fly” after “the little engine that could”. The verbal and the visual may constitute two interacting and even clashing forces, with the verbal directing readers to an explicit intertext while the visual steering them onto other possible paths of interpretation.

Balancing, or rather, rebalancing the power relationship between child and adult readers of picturebooks in the contemporary context of ageism often means deliberately putting more trust in children. Perry Nodelman notes that the verbal text in picturebooks often purports to be childlike, while the visual tends to “undercut that childlike simplicity with a more sophisticated adult view of things”, and he further stresses the view of a hidden observer who sees more details as a “more adult” view (2010, p. 18). With such proficient child readers as Lucy who not only saw numerous pictorial details but adeptly weaved them together into a seamless narrative, it does not stand to empirical test to connect the purported simplicity of the verbal text with a child and a more comprehensive view implied by the pictures with an adult.

The material affordances of a picturebook can be deployed to amplify the points of connection and dialogue in child and adult readers’ responses and a post-age picturebook pedagogy should strive to enrich interpretive and experiential possibilities for both children and adults. First, the material experience of reading a picturebook may help to initiate children into further thinking about abstract notions. Through picturing the books that, in different layouts and positions, shelter the miniature inhabitants, the visual in *Forever* foregrounds the materiality of books as objects that can evoke a tactile and sensuous experience. Such an experience paralleled the readers’ ongoing process of closing and opening the book, fingering and turning the pages, pointing and trailing, going back and forth, and playing it around. The child readers only mentioned books as a physical space, though the foregrounded materiality of books could potentially open up avenues for thinking about books as an abstract space that stores massive information, knowledge and life stories.

A second potential point of connection in child and adult readers’ responses is performativity. The material qualities of picturebooks encourage performativity, which, according to Lawrence Sipe (2008), refers to responses that include page turning, facial expressions, gestures, and sounds. Sipe considers performativity as an important aspect of literary understanding, through which children use texts “as a platform” or “playground for a carnivalesque romp”, “[e]ntering the text world” and “manipulating” it for their own purposes (Sipe, 2008, p. 268). Recent developments in cognitive science and neuroscience point to a fundamental connection between performativity and meaning. Speech and gesture constitute a closely bound-up cognitive system (Iverson and Thelen, 1999). Brain studies using fMRI show that there is some overlap between the brain areas that are activated during language use and manual behaviour (Loring et al., 2000). Moreover, vocal entangled gestures, rooted in biomechanical linkages with respiration, make up an orchestra of movement to generate meaning (Pouw and Fuchs, 2022). Lucy’s narrative of a bustling book world was definitely performative. Yet one is also prompted to reflect on how the performative element may be manifest in adult readers’ responses. The hardwired connection between gesture and speech or meaning aside, though Sophie’s reinvention of another story was not that outwardly performative, it was nevertheless performative in nature because to tell a new story, Sophie had to enter the text world, in this case, visual representations of the fictional world in particular, and adapt the fictional world for her own purpose. How performativity may play out in child and adult readers and how their performativity can be mutually enriching would be a fruitful line of enquiry for further research toward a post-age picturebook pedagogy.

## Conclusion

Several key issues that emerge from the present study may serve as a starting point for developing a post-age picturebook pedagogy:

- Maintain an egalitarian view of child and adult readers;
- Use the picturebooks that may appeal to both children and adults;
- Explore picturebook affordances from multiple perspectives;
- Seek and amplify possible points of connection and dialogue so as to make picturebook reading an enriching activity for both children and adults.

Such an approach arises as a timely response to the contemporary conflict between the discourse of ageism and the prevalence of age-transgressive practices in that it acknowledges and strives to maximise the potential of picturebooks for creating a beneficial and pleasant experience for all involved. Gubar draws on an observation made by Eve Kosofsky Sedgwick—“People are different from each other. It is astonishing how few respectable conceptual tools we have for dealing with this self-evident fact” (quoted in Gubar, 2016, p. 294). Thus, it should not be surprising that drawing up a list of books for children or adults according to their ages, as if they were patients in need of medical prescriptions, may not work. Picturebooks like *Forever* and *Grandpa* provide points of uncertainty, connection and dialogue, opening up various interpretive and experiential opportunities. Reading these picturebooks does not shame adults, nor do they “elevate” children to a sphere that is only accessible to adults, if such a sphere is ever possible.

This article provides some initial thoughts about how a post-age picturebook pedagogy may take shape. The findings should be read with regard to the limited sample size and the individuality and situatedness of the participants’ experiences. The test and modification of the post-age picturebook pedagogy in light of more comprehensive empirical data can be a prospective next study.

## Supplementary information

Appendices I and II referenced in the main text

## Data Availability

The dataset is available from the author upon reasonable request.

## Footnotes

1 Neither of the two selected picturebooks is paginated, which is the standard practice for children’s picturebooks. Thus, I manually count the spread, which refers to the two-facing pages as a picturebook is opened. The first spread includes the title page and its facing page to the left. All the numbered spreads mentioned in this article result from manual counting in this manner.

## References

[CR1] Arizpe E (2021). The state of the art in picturebook research from 2010 to 2020. Language Arts.

[CR2] Baker L, Beall LC, Israel SE, Duffy GG (2009). Metacognitive processes and reading comprehension. Handbook of research on reading comprehension.

[CR3] Beckett S (2009). Crossover fiction: global and historical perspectives.

[CR4] Beckett S (2012). Crossover picturebooks: a genre for all ages.

[CR5] Beckett S (1999). Transcending boundaries: writing for a dual audience of children and adults.

[CR6] Bezemer J, Kress G (2008). Writing in multimodal texts: a social semiotic account of designs for learning. Writ Commun.

[CR7] Burman E (2008). Developments: child, image, nation.

[CR8] Bus AG, Van Ljzendoorn MH (1997). Affective dimension of mother-infant picturebook reading. J School Psychol.

[CR9] Crago H (2014). Entranced by story: brain, tale and teller, from infancy to old age.

[CR10] Creswell JW (2012). Educational research: planning, conducting, and evaluating quantitative and qualitative research.

[CR11] Creswell JW, Poth CN (2017). Qualitative inquiry research design choosing: among five approaches.

[CR12] Desmet M, Pinsent P (2004). The universe of Jimmy: Creating picture books for adults in Taiwan. Books and boundaries: writers and their audiences.

[CR13] Falconer R, Hunt P (2004). Crossover literature. International companion encyclopedia of children’s literature.

[CR14] Falconer R (2009). The crossover novel: contemporary children’s fiction and its adult readership.

[CR15] Fauconnier G, Turner M (2002). The way we think: conceptual blending and the mind’s hidden complexities.

[CR16] Fischer KW (2009). Mind, brain, and education: building a scientific groundwork for learning and teaching. Mind Brain Educ.

[CR17] Flint TK, Adams MS (2018). “It’s like playing, but learning”: Supporting early literacy through responsive play with wordless picturebooks. Language Arts.

[CR18] Gibbs RW (2005). Embodiment and cognitive science.

[CR19] Goswami U (2011). The Wiley–Blackwell handbook of childhood cognitive development.

[CR20] Gubar M (2016). The hermeneutics of recuperation: what a kinship-model approach to children’s agency could do for children’s literature and childhood studies. Jeunesse Young People Texts Cult.

[CR21] Harju LM (2012) Being not alone in the world: exploring reader responses to crossover Books. Dissertation, McGill University

[CR22] Haynes J, Murris K (2017). Intra-generational education: imagining a post-age pedagogy. Educ Philos Theory.

[CR23] Hoffmann L (2018). Crossover. Mehrfachadressierung in text, markt und diskurs.

[CR24] Iverson JM, Thelen E (1999). Hand, mouth and brain. The dynamic emergence of speech and gesture. J Conscious Stud.

[CR25] Johnson M (1987). The body in the mind.

[CR26] Johnston P, Afflerbach P (1985). The process of constructing main ideas from text. Cognit Instr.

[CR27] Kümmerling-Meibauer B (2013). Towards a cognitive theory of picturebooks. Int Res Child Lit.

[CR28] Kümmerling-Meibauer B (2015). From baby books to picturebooks for adults: European picturebooks in the new millennium. Word Image.

[CR29] Lambert MD, Kümmerling-Meibauer B (2018). Picturebooks and page layout. The Routledge companion to picturebooks.

[CR30] Langer JA (1995). Envisioning literature: literary understanding and literature instruction.

[CR31] Larsen SF, Seilman U (1988). Personal remindings while reading literature. Text.

[CR32] Lee N, Motzkau J (2011). Navigating the bio-politics of childhood. Childhood.

[CR33] Levitin DJ (2006). This is your brain on music: the science of a human obsession.

[CR34] Lomax RG, McGee LM (1987). Young children’s concepts about print and reading: toward a model of word reading acquisition. Read Res Q.

[CR35] Loring DW, Meador KJ, Allison JD (2000). Relationship between motor and language activation using fMRI. Neurology.

[CR36] McGilchrist I (2012). The master and his emissary: the divided brain and the making of the Western world.

[CR37] Miall DS (2006). Literary reading: empirical and theoretical studies.

[CR38] Mitchell WJT (1994). Picture theory: essays on verbal and visual representation.

[CR39] Moraru C, Herman D, Jahn M, Ryan ML (2005). Intertextuality. Routledge encyclopedia of narrative theory.

[CR40] Muhinyi A, Hesketh A (2017). Low- and high-text books facilitate the same amount and quality of extratextual talk. First Language.

[CR41] Nikolajeva M (2014). Reading for learning: cognitive approaches to children’s literature.

[CR42] Nikolajeva M, Scott C (2001). How picturebooks work.

[CR43] Nodelman P, Colomer T, Kümmerling-Meibauer B, Silva-Díaz MC (2010). Words claimed: Picturebook narratives and the project of children’s literature. New directions in picturebook research.

[CR44] Ommundsen ÅM, Evans J (2015). Who are these picturebooks for? Controversial picturebooks and the question of audience. Challenging and controversial picturebooks: creative and critical responses to visual texts.

[CR45] Ommundsen ÅM, Haaland G, Kümmerling-Meibauer B (2021). Exploring challenging picturebooks in education: international studies in language and literature learning.

[CR46] Pantaleo S (2012). Middle years students thinking with and about typography in multimodal texts. Lit Learn Middle Years.

[CR47] Pantaleo S (2013). Paneling “matters” in elementary students’ graphic narratives. Lit Res Instr.

[CR48] Paris SG, Hamilton EE, Israel SE, Duffy GG (2009). The development of children’s reading comprehension. Handbook of research on reading comprehension.

[CR49] Pouw W, Fuchs S (2022). Origins of vocal-entangled gesture. Neurosci Biobehav Rev.

[CR50] Prout A (2005). The future of childhood. Towards the interdisciplinary study of children.

[CR51] Shi X (2022). Differences, idiosyncrasies and shared humanity: Reconceptualising crossover literature. Int Res Child Lit.

[CR52] Shiel N, Fowley C, English C, Thouësny S (2013). A second level pictorial turn? The emergence of digital ekphrasis from the visuality of new media. Internet research, theory, and practice: perspectives from Ireland.

[CR53] Shonoda MA (2012). Metaphor and intertextuality: a cognitive approach to intertextual meaning-making in metafictional fantasy novels. Int Res Child Lit.

[CR54] Silva-Díaz MC (2015) Picturebooks, lies and mindreading. Barnelitt Forskningstidsskr 6(1) 10.3402/blft.v6.26972

[CR55] Sipe L (2008). Storytime: young children’s literary understanding in the classroom.

[CR56] Sipe L, McGuire CE (2006). Picturebook endpapers: resources for literary and aesthetic preparation. Child Lit Educ.

[CR57] Smith L (2011). Grandpa green.

[CR58] Smith V, Evans J (2009). Making and breaking frames: crossing the borders of expectation in picturebooks. Talking beyond the page: reading and responding to picturebooks.

[CR59] Stake R (1995). The art of case study research.

[CR60] Sternberg RJ, Goswami U (2011). Individual differences in cognitive development. The Wiley–Blackwell handbook of childhood cognitive development.

[CR61] Thompson C (1995). How to live forever.

[CR62] Turner M, Herman D (2003). Double-scope stories. Narrative theory and the cognitive sciences.

[CR63] Zapata A, Sanchez L, Robinson A (2018). Examining young children’s envisionment building responses to postmodern picturebooks. J Early Child Lit.

