# Supplementary material for: Towards a post-age picturebook pedagogy
Source: Humanit Soc Sci Commun. 2022 Nov 29;9(1):423. doi: 10.1057/s41599-022-01446-4 (PMC9707091; doi:10.1057/s41599-022-01446-4)
Supplement: Supplementary file 1 — Appendices I and II referenced in the main text [file 41599_2022_1446_MOESM1_ESM.docx]

**Appendix I**

The order and wording of these questions were not followed strictly. Rather, the following questions were incorporated subtly into the readers’ thinking-aloud.

*How to Live Forever*

Have you read the book before? If so, is it different this time?

Is there anything you like about the book? What especially catches your attention? Is there anything you dislike about the book? Is there anything that puzzles or surprises you?

Tell me about the story world. Do you remember thinking about the place(s) as you were reading? When you were reading the story, did you see it happening in your imagination? What details in the story helped you see it most vividly? Tell me some of them.

Do you feel as if everything were happening to you, as if you were one of the characters in the story? Or did you feel as if you were an observer, watching what was happening but not part of the action? If you were Peter, would you read the immortal book after what the Ancient Child says? What makes you come to such a decision?

When you first saw this book, even before you read it, what kind of book did you think it was going to be? What made you think this? Now you’ve read it, is it as you expected?

Does this book remind you of other books/stories you’ve known before? Tell me about them. What sticks in your memory most vividly? Can you tell me how they are different, alike, or in any other ways connected?

If I were to give the books to other people, should I give it to people your age or older, or younger? What made you think that I should give it to people your age/the older/the younger?

*Grandpa Green*

Have you read the book before? If so, is it different this time?

Is there anything you like about the book? What especially catches your attention? Is there anything you dislike about the book? Is there anything that puzzles or surprises you?

Tell me about the great-grandfather’s life. Or how would you picture the great-grandfather’s life?

Who is telling the story? Do we know? How do we know? What do you think of the relationship between the great-grandfather and the great-grandchild? Do we know how they may think of each other? How do we know? Do we know how they may feel about each other? How do we know? Think of yourself as a spectator, with whose eyes do you see the story?

When you first saw this book, even before you read it, what kind of book did you think it was going to be? What made you think this? Now you’ve read it, is it as you expected?

Does this book remind you of other books/ stories you’ve known before? Tell me about them. What sticks in your memory most vividly? Can you tell me how they are different, alike, or in any other ways connected?

If I were to give the books to other people, should I give it to people your age or older, or younger? What made you think that I should give it to people your age/the older/the younger?

**Appendix II**

| making connections | - drawing on real-life experience - drawing on literary experience - drawing on world knowledge - drawing on paratextual knowledge - drawing on the system of beliefs and values |
| --- | --- |
| emotional engagement | - deducing others’ (character’s, narrator’s, author’s. etc.) mental states - empathy |
| higher cognitive skills | - reasoning - solving problems (i.e. filling textual gaps) - metacognising |
